# Balance assessment in alcoholic subjects

**DOI:** 10.1590/S1808-86942010000200002

**Published:** 2015-10-19

**Authors:** Paula Michele da Silva Schmidt, Aline Marques Giordani, Angela Garcia Rossi, Pedro Luiz Cóser

**Affiliations:** MSc in Human Communication Disorders, Speech and Hearing Therapist; MSc in Human Communication Disorders, Speech and Hearing Therapist; PhD in Human Communication Disorders, Speech and Hearing Therapist, Professor; PhD in Human Communication Disorders, Otorhinolaryngologist

**Keywords:** alcoholism, labyrinth diseases, dizziness

## Abstract

Alcoholism is a chronic condition, consisting on a state of intoxication caused by the consumption of alcohol beverages. Frequently found signs and symptoms are: gait instability, dizziness and lack of psychomotor coordination, among others.

**Aim:**

to study the influence of alcoholism on postural balance.

**Materials and Methods:**

this is a prospective study. The sample comprehended 32 individuals in the experimental group, members of the Alcoholic Anonymous Group of the city of Santa Maria-RS and 32 non-alcoholic individuals making up the control group. The individuals were submitted to an otorhinolaryngological evaluation, static and dynamic balance study and cerebellar tests, vecto-electronystagmographic evaluation and dynamic posturography.

**Results:**

we noticed that the vectoelectronystagmography was normal for most of the individuals in the experimental group, indicating that the labyrinth and the oculomotor-vestibular pathways were normal and that the balance disorder they presented would stem from the dysfunction in other areas of the central or peripheral nervous system. In the dynamic posturography we noticed that alcoholics who were not drinking presented significant alterations in their postural balance when compared to non-alcoholic individuals.

**Conclusion:**

alcoholic beverages have a deleterious influence on body balance.

## INTRODUCTION

Alcoholism or alcohol dependence is a chronic disease acknowledged by the World Health Organization (WHO). It consists of a state of intoxication caused by alcohol consumption. It affects different people at different rates, according to the person's physical, emotional and psychological characteristics, degree of tolerance to alcohol and type of beverage ingested.

Many authors report that ototoxic agents, such as alcohol, have a negative effect on the balance organs causing dizziness and vertigo^1^, ^2^, ^3^, ^4^.

Body balance is the capacity each human being has to keep oneself standing up or performing body rotation and movement acceleration without oscillation, deviations or falling. In order to have static and dynamic spatial orientation; in other words, balance, we depend on integrations (vestibular, brainstem and cerebellum areas) which allow the Central Nervous System to recognize head movements and positions in relation to body and space. Changes in body balance maintenance can cause symptoms such as vertigo, dizziness and unbalance^5^.

Dizziness can be a very uncomfortable symptom, which drastically impacts all daily activities and does impair quality of life. It is among the most frequent symptoms in the world and it stems from the labyrinth in approximately 85% of the cases^6^.

Studies state that many drugs, including alcohol, cause dizziness as a side effect, negatively impacting motor skills, including tasks with simple reaction time, coordination skills, balance and eye-hand coordination^7^, ^8^, ^9^, ^10^.

Signs and symptoms which are frequently found in alcoholism are anorexia, instability and dizziness, nausea, vomiting, weight loss, fever and abdominal pain, among others^7^.

Some authors report that alcoholism causes premature aging of neuropsychological functions and possible that of the brain^11^.

Considering the little information present in the literature about the relationship between balance and alcoholism, the goal of the present study is to investigate the influence of alcoholism on postural balance.

## MATERIALS AND METHODS

The experimental group was made up of 32 individuals who regularly attended meetings of the Alcoholic Anonymous, 5 females and 27 males, with ages varying between 33 and 73 years.

Afterwards, we gathered those individuals without neurotological complaints, who did not abuse alcohol, with matching age and gender with each individual in the study - to make up the control group (32 individuals).

Alcohol abuse is characterized by drinking large quantities of alcoholic beverages, almost daily, for at least 15 years.

Among the individuals evaluated those who abused drugs other than alcohol and also those who had any ear, nose and/or throat problems were taken off the study.

The first study activity was to give the study individuals the Informed Consent Term, which explained the study's goal, as well as the procedures involved in all the evaluations; and it requested the person's authorization by means of his/her signature in order to participate in the study.

The individuals were submitted to otolaryngological exam, interview, dynamic and static balance tests, dynamic posturography test and vector electro-nystagmography.

All 32 individuals from the experimental group underwent full ENT exam, interview and dynamic posturography evaluation. For static and dynamic balance tests and vector electronystagmography, only 29 individuals from the experimental group participated, that is, 3 individuals did not come to the clinic in order to undergo the tests.

During the interview we collected information regarding the use of alcohol and the vestibulo-cochlear system of the individuals. It was carried out in order to place the individual as an alcoholic and also to rule out any interference on the vestibulo-cochlear system other than alcohol. The individuals from the control group were interviewed only regarding the issues associated with the cochleovestibular apparatus.

The ENT evaluation was done by an otolaryngologist, with the goal of ruling out any ear, nose and/or throat disorder.

To look for exclusion criterion, and also for middle ear evaluation, the individuals were submitted to basic audiologic evaluation in a sound booth, made up of Threshold Tonal Audiometry, Speech Recognition Threshold (SRT), Speech Recognition Index (SRI) and Acoustic Immittance Measures. As far as the hearing evaluation goes, we used the following equipment: a Fonix FA-12 audiometer, TDH-39 phones and MX-41 pad, a SONY D-11 CD Player SN 9161852 coupled to the audiometer and an Interacoustic AZ7 middle ear analyzer, with a TDH-39 head phone and MX-41 pad, with a 220 Hz sound at 70 dB, both calibrated according to ISO 389–1991 standard.

In order to assess the static and dynamic balance and cerebellar function, we used the following tests described by Mangabeira-Albernaz & Ganança (1976): Gait Test, Romberg and Romberg-Barré, Unterberger test, Extended Arms Test, diadochokinesia and dysmetria tests.

Dynamic posturography (DP) was carried out through the Foam-Laser Dynamic Posturography (FLP) method, proposed by Castagno^12^. It is based on a very simple technique to assess the individual's sensory organization - SOT (Sensorial Organization Test). The patient is put within a 1m^2^ x2m-high booth, and with a visual image made up of blue and beige 10cm stripes. This visual image with stripes aims at creating a visual conflict. At the patient's side, at the height of his hip on the right side, in the gravity center, we place a laser pointer pointing to a scale in centimeters attached to the ceiling, in a vertical plane. Six readings are carried out (SOT I, II, III, IV, V and VI), each one within 20s duration.

In order to carry out the vestibular test, we used a computerized Vector Electronystagmography System - SCV 5.0, updated from system SCV 4.0, proposed by Castagno^12^. The following tests became part of the vestibular test: Eye Movement calibration, spontaneous nystagmus, semispontaneous nystagmus, optokinetic nystagmus, pendular tracking, decreasing pendular rotational test (DPRT) and caloric test.

This study is in accordance with the ethical principles found in the Helsinki Declaration (1964, revised on 1975, 1983, 1989, 1996 and 2000), from the World Medical Association, besides complying with the specific legislation from the country. This study is registered in the Ethics in Research Committee of the Institution where it was done, under protocol # 23081.003726/2006-41.

### Data Analysis

In order to assess the possible differences between the experimental and control groups regarding the Dynamic Posturography variables, we used the Kruskal-Walis non-parametric test, in which we used a 5% significance level, in other words, p<0.05, marking the significant values with an asterisk.

## RESULTS

For a clearer presentation, we broke the data down in two parts:

PART I: results obtained from the vestibular test in the 29 individuals from the experimental group (group E).

On Table 1 we find the results obtained from the Dynamic and Static balance regarding the individuals in the experimental group. We noticed that from the 29 individuals evaluated, 26 (89.66%) had a normal response, and 3 (10.34%) had an altered response on the Romberg test. Regarding the Romberg-Barré test, 16 (55.17%) presented normal responses and 13 (44.83%) had an altered response. We also observed that from the 29 individuals evaluated, 11 (37.93%) had altered Untemberg tests and 18 (62.07%) did not show alterations in this test. In the gait test analysis, 16 (55.17%) did not show alteration and 13 (44.83%) had an altered response.

**Table 1:** Results obtained from the Static and Dynamic Balance tests done on Group E individuals.

DYNAMIC AND STATIC BALANCE						
	With alteration		Without alteration		total	
	N	%	N	%	N	%
Romberg	3	10,34	26	89,66	29	100,00
Romberg-Barré	13	44,83	16	55,17	29	100,00
Untemberg	11	37,93	18	62,07	29	100,00
Gait	13	44,83	16	55,17	29	100,00

In the Extended Arms Test, in the diadochokinesia and dysmetria tests in the experimental group individuals, results were within normal standards.

The experimental group individuals did not show alterations in the vertical nor horizontal calibration, and the same happened to the Optokinetic Nystagmus, the Spontaneous Nystagmus and Semi-Spontaneous nystagmus.

In the Horizontal Tracking Test, of the 29 experimental group individuals, 27 had type I trace, 1 individual had a Type II and another one showed a Type III trace.

Regarding the presence or absence of per-rotational nystagmus (Table 2) we noticed that of the 29 individuals evaluated, 27 (93.10%) had symmetrical per-rotational nystagmus, and only 2 (6.90%) had alterations.

**Table 2:** Results obtained in the Per-Rotational Nystagmus study done on Group E individuals.

PER-ROTATIONAL NYSTAGMUS		
	N	%
symmetrical	27	93,10
Altered	2	6,90
Total	29	100,00

In the study regarding the presence or absence of post-caloric nystagmus (Table 03) we noticed that of the 29 individuals evaluated, 23 (79.31%) presented normal reflexes, 4 (13.79%) had changes in this test and 2 (6.90%) did not perform the test.

**Table 3:** Results obtained in the Post-Caloric Nystagmus test done on Group E individuals.

POST-CALORIC NYSTAGMUS		
	N	%
Normal	23	73,31
Altered	4	13,79
Did not do	2	6,90
Total	29	100,00

On Table 4 we notice that, at the end of the vector electronystagmography, of the 29 individuals evaluated, 25 (86.21%) had a normal test, 3 (10.34%) individuals had peripheral vestibular syndrome and 1 (3.45%) individual presented central vestibular syndrome.

**Table 4:** Results obtained in the conclusion of the vector electronystagmography test on Group E individuals.

VECTOR ELECTRONYSTAGMOGRAPHY TEST		
	N	%
Normal	25	86,21
Peripheral VS	3	10,34
Central VS	1	3,45
Total	29	100,00

PART II: results obtained from the dynamic posturography test in the 32 individuals from the experimental group and the 32 from the control group.

As we can see on Table 5, the dynamic posturography showed a statistically significant difference in all SOT situations.

**Table 5:** Results from the minimum, maximum, mean and standard deviation values of Dynamic Posturography from to Groups C and E.

DYNAMIC POSTUROGRAPHY									
	Minimum		Maximum		Mean		Standard Deviation		P
	G C	G E	G C	G E	G C	G E	G C	G E	
SOT I	91,52	73,72	95,81	95,81	86,32	40,32	2,2295	12,3328	<0,01
SOT II	80,92	1,07	93,23	90,74	85,39	67,68	2,9230	15,3841	<0,01
SOT III	77,22	16,96	88,71	90,74	83,01	65,47	2,8766	17,6910	<0,01
SOT IV	72,95	57,49	91,11	90,93	81,73	73,35	4,5931	9,7590	<0,01
SOT V	54,86	0,32	86,46	81,50	65,88	46,46	7,6059	22,9704	<0,01
SOT VI	46,61	-13,40	79,70	77,78	57,89	40,51	6,6332	24,0334	<0,01
Mean	71,51	37,03	214,00	74,47	81,88	61,20	24,8376	9,1423	<0,01

We noticed a highly significant difference between the mean values of the results from the investigated groups (p < 0.01).

## DISCUSSION

Analyzing the answers from the individuals in the Romberg test (Table 1), we noticed that of the 29 individuals evaluated, 26 (89.66%) had normal responses and 3 (10.34%) had an altered response. The number of individuals without alterations in the test was greater than the number of individuals with alteration.

In the Romberg-Barré test (Table 1), of the 29 individuals evaluated, 16 (55.17%) had normal response and 13 (44.83%), had an altered response. We noticed a significant increase in the number of altered results in this test when compared to the Romberg test, which can be explained by the fact that the Romberg-Barré test is an augmented Romberg Test, thus requiring better body balance.

We also noticed that of the 29 individuals evaluated, 11 (37.93%) had altered Untemberg test and 18 (62.07%) did not have alterations in this test (Table 1).

In analyzing the Gait test (Table 1), of the 29 individuals evaluated, 16 (55.17%) did not have alterations and 13 (44.83%) had altered responses. The number of alterations in this test was high, revealing individual incapacity regarding motor coordination, in agreement with Giron^13^, who reported that alcohol-induced neurological complications involve numerous aspects, among them we have: alcoholic intoxication, withdrawal syndrome, and others: movement and gait lack of coordination, instability and dizziness.

The instability of the individuals evaluated can be explained by Ledin & Ödkvist^14^ who observed that individuals who had been chronic alcoholics had balance impairment, suggesting that this may be due to a possible general complication, a cerebellar degeneration, still rarely diagnosed in the clinical setting. It is this very degeneration that causes severe gait instability.

These are complementary importance tests because they offer additional topodiagnosis information, vis-à-vis other data from the vestibular exam, and never alone, in agreement with Caovilla, Ganança, Munhoz, Silva & Settanni^15^ who reported that these tests show dynamic and static balance alterations, the damaged side and the lesion site.

In the cerebellar tests: extended arms test, dysmetria and diadochokinesia test, the individuals assessed did not show alterations.

In the pendular tracking test, the Type I trace prevailed, indicating a normal test. One individual presented a type III trace which is also associated with normal individuals, and another had a Type III trace which can be seen in individuals with central or peripheral vestibular disorders.

We did not find alterations in the Optokinetic Nystagmus in any of the individuals assessed in this study. According to Ganança, Caovilla, Munhoz, Silva & Settanni^15^, optokinetic nystagmus is less sensitive than tracking under attention and the use of medication, and also less sensitive in its capacity to detect alterations in central localization, which can explain the number of alterations found in the pendular tracking test and not in the optokinetic nystagmus test - in disagreement with Nieschal et al.^16^, who reported that many investigators have reported alcohol effects on the oculomotor system.

In the test regarding the presence or absence of the per-rotational nystagmus (Table 2) it was noticed that of the 29 individuals evaluated, 27 (93.10%) had symmetrical per-rotational nystagmus, and only 2 (6.90%) had alterations.

In the study regarding the presence or absence of alterations in the post-caloric nystagmus test (Table 3), we observed that of the 29 individuals assessed, 23 (79.31%) had normal reflexes, 4 (13.79%) had alterations in such test and 2 (6.90%) did not undergo the test.

On Table 4, we observed that at the conclusion of the vector electronystagmography, of the 29 individuals assessed, 25 (86.21%) had a normal test, 3 (10.34%) individuals had peripheral vestibular syndrome, and the topographic diagnosis of peripheral vestibular disease in the three cases was characterized by the absence of pathognomonic signs of central lesion and the presence of alterations in relation to the normal standard, thus making up a diagnosis of exclusion. One (3.45%) individual had a central vestibular syndrome. Mascari, Zeigelboim, Fukuda, Anadão & Ganança^17^, in their study with patients with vestibulocochlear disorders caused by alcohol also found, among the vestibular test, alterations which showed lesions on their central vestibular system.

We did not observe a significant number of alterations in the assessment of the vestibular system through computerized vector electronystagmography. These findings can be explained by the fact that the individual differences in the postural response regarding alcohol can be genetic related, depend on the family history of alcoholism and daily consumption levels. All these differences may have impaired the neurotological diagnosis.

It is important to stress that it is possible to find patients with typical vertigo which can, at a certain point, yield normal results in exams, as it is possible that a vestibular test in a patient without significant vestibular involvement, would not mean that its vestibular system is truly normal^18^.

Numerous authors showed that the effects of alcohol abuse over a long period of time on balance are well known. Chronic alcoholics have numerous difficulties in simple balance tests and also when walking, manifesting an ataxic gait with a broad base. A mild effort for chronic alcoholics with cerebellar lesion may cause them to lose balance, showing their lack of correction mechanisms. In this study we noticed that alcoholic individuals did not show alterations in their vector electronystagmography, very likely because they had problems in other areas of the central or peripheral nervous system, and not the vestibular organs. Bellé, Sartori & Rossi^19^ in a study with alcoholics noticed that the alterations seen in the vector electronystagmography were more evident in the experimental group individuals when compared to those from the control group, considering the experimental group with individuals between 33 and 49 years of age as well as for the experimental group with individuals between 50 and 70 years old, and the latter was in larger number. In the specialized literature, authors reported results very similar to the ones found in the present investigation, stating that ototoxic agents, such as alcohol have a negative effect on the vestibular organs, causing dizziness and vertigo^1^^,^^2^^,^^3^^,^^4^.

As we can notice on Table 5, dynamic posturography, held by means of the Foam-Laser Dynamic Posturography presented a statistically significant difference in all SOT situations.

Groups E and C individuals in the SOT I had statistically significant different performance among the groups studied, even if it is a condition deemed simple, in which the individuals remain standing up, looking ahead. Subclinical cerebellar body tremor is a possible cause for balance performance reduction, obviously chronic alcoholics seem to have pronounced problems of postural stability^14^.

We can see on Table 5 that under SOT II condition, which is the same previous condition, however with the eyes closed, there was also statistically significant difference between the groups. Concerning Liguori, D'agostino, Dworkin, Edwards & Robinson^20^ an intoxication caused by alcohol abuse produces an intense balance reduction, and studies have found a balance reduction also caused by an absent visual system. Another fact which can explain such alteration is this increased body antero-posterior balance with lack of vision which, according to Ledin & Ödkvist^14^ and Tianwu Watanabe, Asai, Shimizu, Takada & Mizukoshi^21^, is correlated with cerebellum anterior lobe atrophy.

The results found on SOT I and SOT II are in agreement with those from Diener, Dichgans, Bacher, Hülser & Liebach^22^ who observed that PD revealed a significant increase in body balance, not only with the eyes closed, but also with the eyes open, revealing a lack of compensation for the ataxia induced by ethanol by means of visual stabilization.

On SOT III - in which the individual has altered vision, there also was a statistically significant difference between the groups tested. One possible explanation for this finding would be the functional impact of the peripheral and/or central vestibular abnormalities in the balance of patients which can be categorized as an inability to suppress/cancel the influence of inaccurate visual information^23^.

On SOT IV, individuals remain standing up, with their feet together, keeping themselves up on 10cm of medium density mobile foam, looking straight ahead. Under such condition, it is the proprioception that is altered. We observed a statistically significant difference between the groups. Such alteration can stem from a disorder on central vision integration and vestibular information. For Liguori, D'agostino, Dworkin, Edwards & Robinson^20^ in alcohol abuse intoxication the individual is more likely to have a general weakening of the CNS than a specific sensorial abnormality. There have been reports of multiple functions weakening, and the vestibular result is the most marked one, especially when somatosensory information is inaccurate.

As to the performance of individuals from groups E and C in the SOT V, in which the individuals remained standing up, having their feet together on 10cm of medium density mobile foam, with eyes closed, there was also a statistical difference between the groups. Such alteration is due to a lack of ability to use the vestibular information. Tianwu, Watanabe, Asai, Shimizu, Takada & Mizukoshi^21^, in their studies, also found postural instability on the SOT V condition, such a condition in which the individual has lack of vision and altered proprioceptive information, then, it is the vestibular system competing for an important function in the maintenance of postural balance.

On SOT VI, when vision and proprioception are altered, there was also a statistically significant difference, and the explanation for this is the very combination of the lack of skill to use vestibular information and the lack of skill to suppress/cancel the influence of inaccurate visual information.

In studies by Nashner & Peters^23^, patients with peripheral vestibular disorders showed results of abnormal balance, especially under SOT V and SOT VI conditions, which require normal vestibular function in order to maintain stability in the position.

Parker^24^ suggests that patients with severely reduced vestibular function are capable of compensating for a loss of proprioceptive input or visual input, but find difficulties in compensating for a loss in which both the proprioceptive and visual sensorial information are altered and had more difficulties with sensorial distortions than with the loss of sensorial information. Such considerations can explain the alterations found in SOT V and in SOT VI.

Goebel, Dunham, Rohrbaugh, Fischel & Stewart^25^ reported that PD procedures showed a high level of sensitivity regarding alcohol, especially when the proprioceptive function was distorted or absent. Such findings suggest that the weakening derives from vestibular disorders and they can be compensated in a minimal way by an intact vision. The results found in our study, using the Foam-Laser Dynamic Posturography corroborate the literature studied.

Mendonça, Rossi, Flores & Teixeira^26^ assessed 30 male individuals with ages between 32 and 72 years, 10 reported they used alcohol only (GA), 10 used alcohol and/or illicit drugs (GAD) and 10 healthy individuals reported they did not chronically abuse alcohol and also had never used illicit drugs - these formed the control group (GC).

We noticed that the individuals from the GA obtained balance values lower than those from the GC in the SOT I, II, IV and V, and the GAD individuals obtained values lower than GC only on SOT VI, and in the SOT III and VI the three groups showed similar values. Considering the GA - where we found values lower than those from the GC in SOT I, II, IV and V, the results were associated to the fact that the proprioceptive and vestibular systems may be affected by alcohol intoxication and the visual system can act by compensating the deficiency in these systems, having seen that when vision was taken off, the individuals obtained the worst balance values.

The final SOT average from groups E and C also showed statistically significant differences. One explanation for such finding is that alcohol reduces the nerve action potential current and can be considered as a means for central depression, causing delays in reflexes and reactions, suggesting that signals coming from the labyrinth are late and interpreted as a disorder, as well as disorders from the oculomotor system, which indicate alterations in the semi-circular canals - caused by alcohol^27^. According to Lima^28^ chronic alcoholism can cause brain atrophy, compromising the mental, physical and social performance of these individuals.

Through Dynamic Posturography, researchers have studied the vestibulo-spinal aspect of the vestibular function, which is usually neglected on the evaluation of the alcohol-caused intoxication and observed a significant impact on body balance. The area of increased balance studied expanded with the eyes open as well as with the eyes closed, revealing an inadequate compensation of ethanol-induced ataxia by visual stabilization^22^.

Ledin & Odkvist^14^ studied 11 chronic alcoholic men aged between 44–65 years through Dynamic Posturography, who had been drinking alcoholic beverages for 20 years in average and had abstinence time between 1–20 years (mean time of 7 years). They concluded that the abnormal patterns found in the dynamic posturography and in other tests suggest alcohol-induced cerebellar lesion and they believe that Dynamic Posturography is a valuable test to assess unbalance in chronic alcoholics, even during abstinence.

These same authors in 1991, studied 13 healthy volunteer men, aged between 21–42 years (mean of 27 years) through Dynamic Posturography before and after alcohol ingestion and concluded that DP is able to detect the effects of alcoholic beverages on the dynamic and static balance, and the test conditions in the absence of vision seemed to be the most sensitive.

A study assessed 13 alcoholic men through DP before and after drinking alcoholic beverages, and they concluded that the test is able to detect the effects of alcohol both on the static and the dynamic balance, and test conditions without vision (TOS II and TOS V) seemed to be more sensitive to balance disorders^27^.

In our study we could notice a greater sensitivity of dynamic posturography when compared to vector electronystagmography as to the detection of balance alterations allegedly caused by alcohol ingestion. Such finding is in agreement with the literature. Electronystagmography has been for many years the main clinical test used to assess the vestibular function, and DP is a totally different method used to measure balance in comparison to traditional tests. According to Parker^24^, DP is a method of balance quantification and description in a static position and balance in response to changes in sensorial inputs and movement platforms, and this set of tests is more useful, showing and quantifying balance abnormalities instead of finding etiological diagnosis. It may show abnormalities when other tests are normal and in combination with electronystagmography and rotational tests it can pinpoint the lesion.

Asai, Watanabe, Ohashi & Mizukoshi^29^, in their routine balance assessment of their vestibular patients, have occasionally found patients complaining of dizziness or vertigo, although the electronystagmography did not show abnormal findings, thus showing that DP could be useful to detect vestibular dysfunctions in certain cases.

According to Ledin & Ödkist^30^, with a sensitive method such as DP, balance disorders will be detected early on and with greater sensitivity, better than with the traditional balance evaluation methods.

Dynamic posturography provides new information on the vestibular patient situation and the vestibular compensation, since it can assess the vestibulo-spinal function separately from the visual and/or proprioceptive information^29^^,^^31^.

Computerized dynamic posturography is very expensive, which makes its acquisition impossible for most of the institutions. Now, Foam-Laser Posturography is a fast, inexpensive and relatively easy method to use, and it can provide a good evaluation of the body balance triad^12^.

In this study we can notice that the alcoholics, even during abstinence, had significant postural balance alterations using the Foam-Laser Posturography, when compared to non-alcoholic individuals.

## CONCLUSION

After doing the static and dynamic tests, computerized vector electronystagmography and dynamic posturography in individuals members of an AA group, it was possible to notice that:

– Dynamic Posturography proved efficient to detect balance alterations in alcoholic individuals.
– Computerized vector electronystagmography proved normal in most of the individuals of the experimental group, indicating that the labyrinth and the vestibulo-oculomotor pathways were normal and the balance disorder they had would stem from dysfunctions in other areas of the central or peripheral nervous system.

Thus, after doing this study and considering the experimental conditions employed, it was possible to conclude that alcohol has a deleterious influence on postural balance.
